# Crystal structure of 2-[4(*E*)-2,6-bis­(4-chloro­phen­yl)-3-ethyl­piperidin-4-yl­idene]acetamide

**DOI:** 10.1107/S2056989015018666

**Published:** 2015-10-10

**Authors:** K. Priya, K. Saravanan, S. Selvanayagam, S. Kabilan

**Affiliations:** a Department of Chemistry, Annamalai University, Annamalainagar, Chidambaram 608 002, India; b PG & Research Department of Physics, Government Arts College, Melur 625 106, India

**Keywords:** crystal structure, piperidine derivatives, N—H⋯O hydrogen bonds, N—H⋯π inter­actions

## Abstract

In the title piperidine derivative, C_21_H_22_Cl_2_N_2_O, the piperidine ring adopts a chair conformation. The chloro­phenyl rings are oriented at an angle of 45.59 (14)° with respect to each other. In the crystal, mol­ecules are linked *via* N—H⋯O hydrogen bonds, forming *C*(4) chains along [100]. The chains are linked by C—H⋯O hydrogen bonds, forming sheets parallel to the *ab* plane. Within the sheets, there are N—H⋯π inter­actions present. The crystal studied was refined as an inversion twin.

## Related literature   

For background to piperidienes, their properties and syntheses, see: Deopura *et al.* (2008 ▸); Greenberg *et al.* (2000 ▸); Johnsson (2004 ▸); Katritzky *et al.* (1989 ▸); Kornblum & Singaram (1979 ▸); Moorthy & Singhal (2005 ▸); Prostakov & Gaivoronskaya (1978 ▸); Yu *et al.* (2002 ▸); Zabicky (1970 ▸).
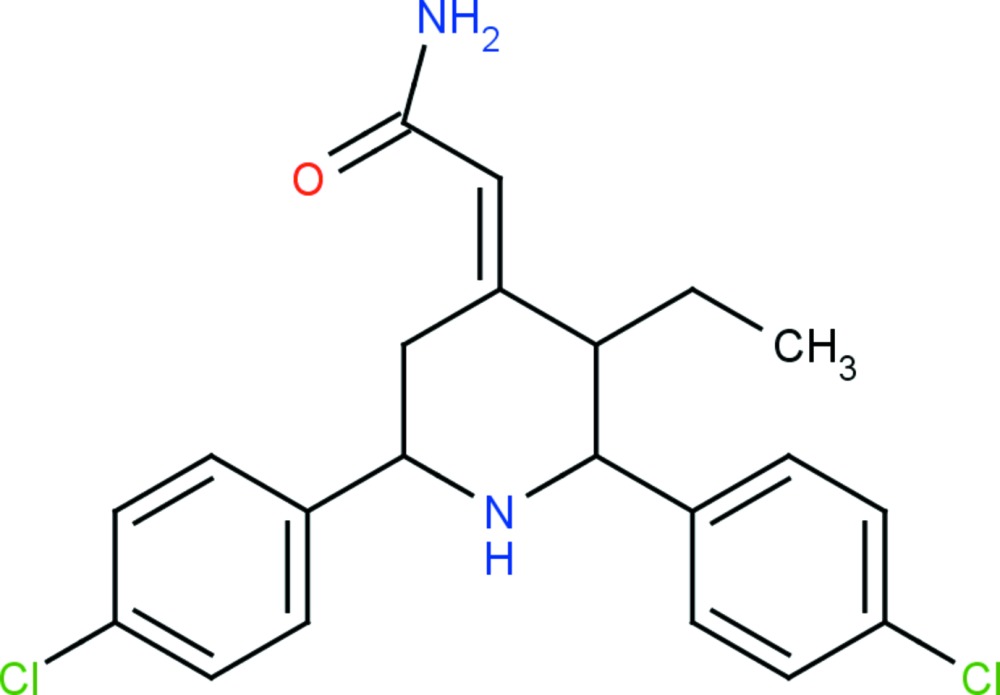


## Experimental   

### Crystal data  

C_21_H_22_Cl_2_N_2_O
*M* *_r_* = 389.30
Orthorhombic, 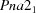

*a* = 8.3293 (2) Å
*b* = 12.2924 (3) Å
*c* = 19.3226 (4) Å
*V* = 1978.39 (8) Å^3^
*Z* = 4
Mo *K*α radiation
μ = 0.34 mm^−1^
*T* = 296 K
0.22 × 0.20 × 0.18 mm

### Data collection  

Bruker SMART APEX CCD area-detector diffractometer
31733 measured reflections
4427 independent reflections
4220 reflections with *I* > 2σ(*I*)
*R*_int_ = 0.022

### Refinement  

*R*[*F* ^2^ > 2σ(*F* ^2^)] = 0.035
*wR*(*F* ^2^) = 0.097
*S* = 1.04
4427 reflections
248 parameters
4 restraints
H atoms treated by a mixture of independent and constrained refinement
Δρ_max_ = 0.40 e Å^−3^
Δρ_min_ = −0.30 e Å^−3^
Absolute structure: Refined as an inversion twin
Absolute structure parameter: 0.37 (7)

### 

Data collection: *SMART* (Bruker, 2001 ▸); cell refinement: *SAINT* (Bruker, 2001 ▸); data reduction: *SAINT*; program(s) used to solve structure: *SHELXS97* (Sheldrick, 2008 ▸); program(s) used to refine structure: *SHELXL2014*/7 (Sheldrick, 2015 ▸); molecular graphics: *ORTEP-3 for Windows* (Farrugia, 2012 ▸) and *PLATON* (Spek, 2009 ▸); software used to prepare material for publication: *SHELXL2014*/7 and *PLATON*.

## Supplementary Material

Crystal structure: contains datablock(s) I, global. DOI: 10.1107/S2056989015018666/su5218sup1.cif

Structure factors: contains datablock(s) I. DOI: 10.1107/S2056989015018666/su5218Isup2.hkl

**Fig. 1.**
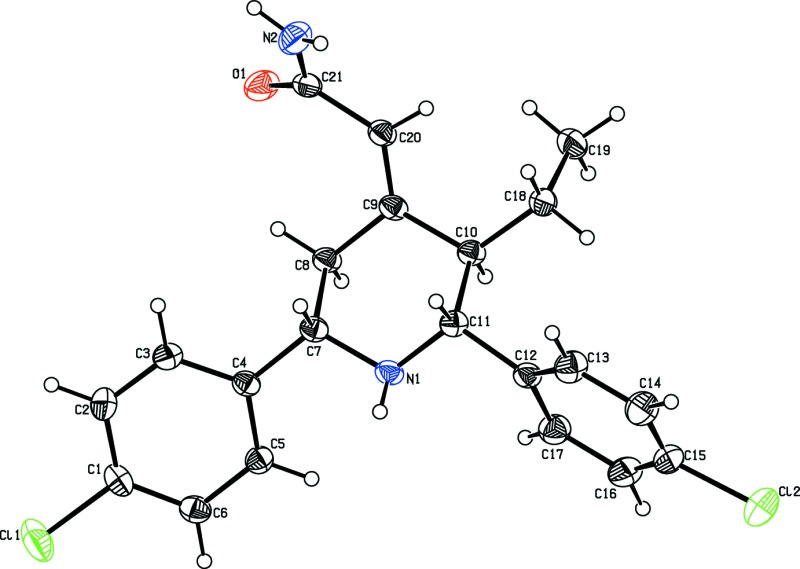
The molecular structure of the title compound, with atom labelling. Displacement ellipsoids are drawn at the 30% probability level.

**Fig. 2.**
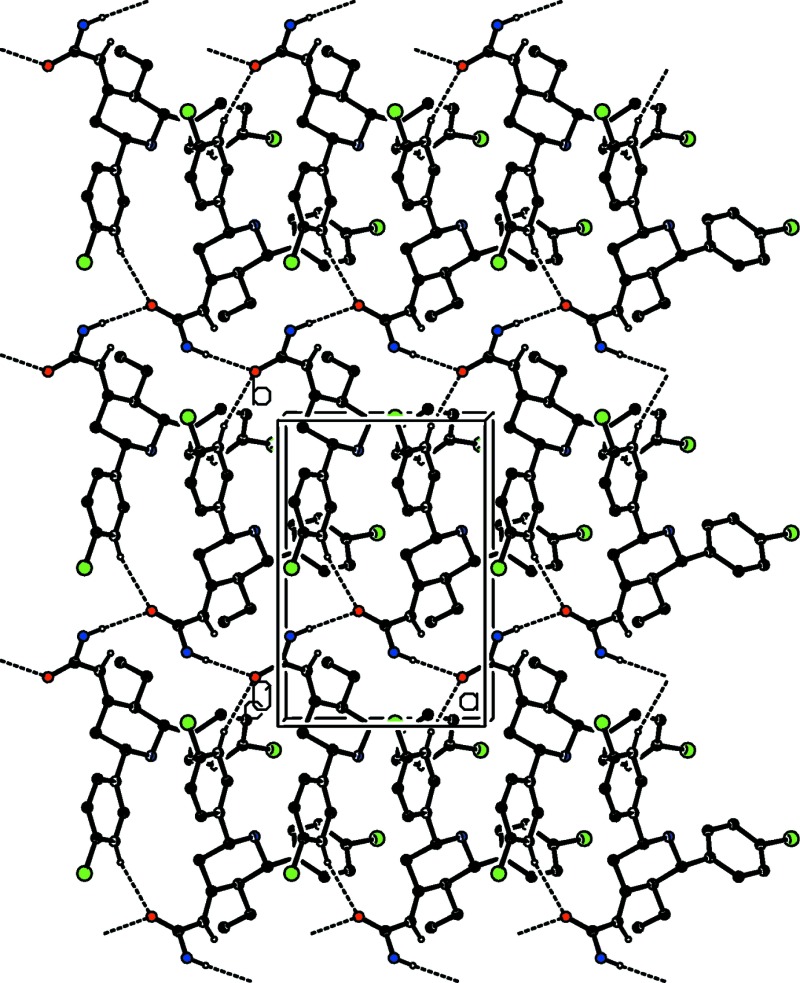
Crystal packing of the title compound, viewed along the c axis. The N—H···O and C-H···O hydrogen bonds are shown as dashed lines (see Table 1). For clarity H atoms not involved in these hydrogen bonds have been omitted.

. DOI: 10.1107/S2056989015018666/su5218fig1.tif
The mol­ecular structure of the title compound, with atom labelling. Displacement ellipsoids are drawn at the 30% probability level.

c . DOI: 10.1107/S2056989015018666/su5218fig2.tif
Crystal packing of the title compound, viewed along the *c* axis. The N—H⋯O and C-H⋯O hydrogen bonds are shown as dashed lines (see Table 1). For clarity H atoms not involved in these hydrogen bonds have been omitted.

**Fig. 3.**
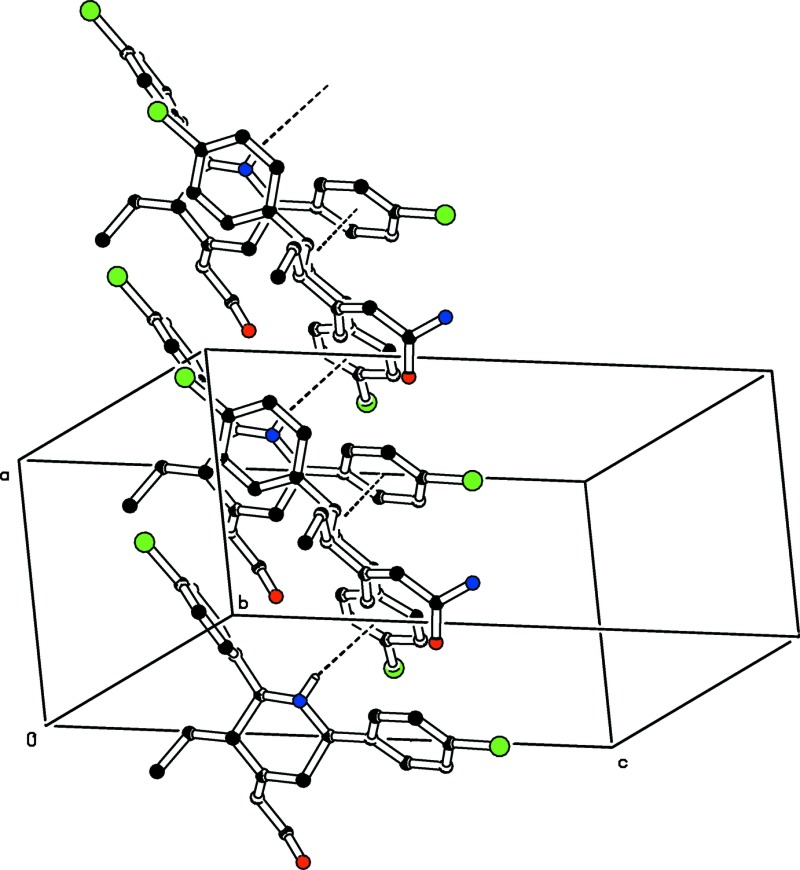
Crystal packing of the title compound, showing the N—H···π interactions as dashed lines (see Table 1). For clarity H atoms not involved in these interactions have been omitted.

. DOI: 10.1107/S2056989015018666/su5218fig3.tif
Crystal packing of the title compound, showing the N—H⋯π inter­actions as dashed lines (see Table 1). For clarity H atoms not involved in these inter­actions have been omitted.

CCDC reference: 1064003

Additional supporting information:  crystallographic information; 3D view; checkCIF report

## Figures and Tables

**Table 1:** Hydrogen-bond geometry (, ) *Cg* is the centroid of the C1C6 ring.

*D*H*A*	*D*H	H*A*	*D* *A*	*D*H*A*
N2H2*NB*O1^i^	0.82(1)	2.17(2)	2.973(3)	165(4)
C6H6O1^ii^	0.93	2.55	3.454(3)	163
N1H1*N* *Cg* ^ii^	0.82(1)	2.85(4)	3.626(2)	157(3)

Symmetry codes: (i) 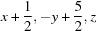; (ii) 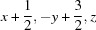.

## supplementary crystallographic information

### S1. Chemical context

The significance of piperidin-4-one as inter­mediates in the synthesis of a range of physiologically active compounds have been reviewed by (Prostakov & Gaivoronskaya, 1978). 4-piperidone derivatives were found to be superior raw materials for preparation of analgesics (Yu *et al.*, 2002). The amide bond is one of the most important functional groups in current chemistry since amides are multipurpose synthetic inter­mediates used in the manufacture of several pharmacological products, polymers, detergents, lubricants, and drug stabilizers, as well as key structural motifs present in numerous natural products (Zabicky, 1970; Greenberg *et al.*, 2000; Deopura *et al.*, 2008; Johnsson, 2004). Usually, amides have been synthesized by the hydration of nitriles, catalyzed by strong acids (Moorthy & Singhal, 2005) and bases (Kornblum & Singaram, 1979; Katritzky *et al.*, 1989). In view of the many inter­esting applications of piperidine derivatives we synthesized the title compound and report herein its crystal structure.

### S2. Structural commentary

The molecular structure of the title compound is illustrated in Fig. 1. The piperidine ring adopts a chair conformation: puckering parameters q_2_ = 0.007 (3) Å, q_3_ = -0.609 (3) Å, Q_T_ = 0.609 (3) Å, and φ = -175.0 (1)°. Atoms C10 and C7 deviate by 0.724 (3) and -0.714 (3) Å, respectively, from the mean plane through the remaining four atoms. The chlorine atoms, Cl1 and Cl2, deviate by -0.019 (1) and 0.135 (1) Å, respectively, from the chloro­phenyl rings (C1—C6) and (C12—C17) to which they are attached. The two chloro­phenyl rings (C1—C6 and C12—C17) are oriented at a dihedral angle of 45.59 (14)°, and are inclined to the mean plane through the piper­idene ring by 76.32 (13) and 46.27 (12) °, respectively.

### S3. Supra­molecular features

In the crystal, moleculesare linked *via* N—H···O hydrogen bonds into C(4) chains propagating along [100] (Table 1 and Fig. 2). The chains are linked by C—H···O hydrogen bonds forming sheets parallel to the *ab* plane (Table 1 and Fig. 2). Within the sheets there are N—H···π inter­actions present (see Table 1 and Fig. 3).

### S4. Synthesis and crystallization

The title (2,6-di­aryl­piperidin-4-yl­idene)aceto­nitrile was refluxed with a few drops of diluted Sulphuric acid for 30-45 mins. After completion of the reaction (monitored by TLC) the mixture was neutralized with saturated sodiumbicarbonate solution, until the disappearance of brisk effervescence. After the solid that appeared was filtered and dried. This crude product mass was purified by column-chromatography over silica-gel (100–200 mesh) using petroleum ether and ethyl­acetate (25%) as eluent to give the title compound. Suitable colourless block-like crystals were obtained by slow evaporation of a solution of the title compound in ethanol at room temperature.

### S5. Refinement

Crystal data, data collection and structure refinement details are summarized in Table 2. Atoms H1N, H2NA and H2NB were located from a difference Fourier map and freely refined. The remaining H atoms were positioned geometrically and treated as riding on their parent C atoms: C—H = 0.93-0.98 Å with U_iso_(H) = 1.5U_eq_ (C) for methyl H atoms and 1.2U_eq_(C) for other H atoms.

### Crystal data

**Table:**

C_21_H_22_Cl_2_N_2_O	*D*_x_ = 1.307 Mg m^−^^3^
*M**_r_* = 389.30	Mo *K*α radiation, λ = 0.71073 Å
Orthorhombic, *P**n**a*2_1_	Cell parameters from 24148 reflections
*a* = 8.3293 (2) Å	θ = 2.2–27.4°
*b* = 12.2924 (3) Å	µ = 0.34 mm^−^^1^
*c* = 19.3226 (4) Å	*T* = 296 K
*V* = 1978.39 (8) Å^3^	Block, colourless
*Z* = 4	0.22 × 0.20 × 0.18 mm
*F*(000) = 816	

### Data collection

**Table:**

Bruker SMART APEX CCD area-detector diffractometer	*R*_int_ = 0.022
Radiation source: fine-focus sealed tube	θ_max_ = 27.5°, θ_min_ = 2.0°
ω scans	*h* = −10→10
31733 measured reflections	*k* = −15→15
4427 independent reflections	*l* = −25→23
4220 reflections with *I* > 2σ(*I*)	

### Refinement

**Table:**

Refinement on *F*^2^	Secondary atom site location: difference Fourier map
Least-squares matrix: full	Hydrogen site location: mixed
*R*[*F*^2^ > 2σ(*F*^2^)] = 0.035	H atoms treated by a mixture of independent and constrained refinement
*wR*(*F*^2^) = 0.097	*w* = 1/[σ^2^(*F*_o_^2^) + (0.0559*P*)^2^ + 0.4463*P*] where *P* = (*F*_o_^2^ + 2*F*_c_^2^)/3
*S* = 1.04	(Δ/σ)_max_ < 0.001
4427 reflections	Δρ_max_ = 0.40 e Å^−^^3^
248 parameters	Δρ_min_ = −0.29 e Å^−^^3^
4 restraints	Absolute structure: Refined as an inversion twin
Primary atom site location: structure-invariant direct methods	Absolute structure parameter: 0.37 (7)

### Special details

**Table:**

Geometry. All esds (except the esd in the dihedral angle between two l.s. planes) are estimated using the full covariance matrix. The cell esds are taken into account individually in the estimation of esds in distances, angles and torsion angles; correlations between esds in cell parameters are only used when they are defined by crystal symmetry. An approximate (isotropic) treatment of cell esds is used for estimating esds involving l.s. planes.
Refinement. Refined as a 2-component inversion twin

### Fractional atomic coordinates and isotropic or equivalent isotropic displacement parameters (Å^2^)

**Table:**

	*x*	*y*	*z*	*U*_iso_*/*U*_eq_	
Cl1	0.04419 (14)	0.50344 (7)	0.45059 (5)	0.0738 (3)	
Cl2	0.93853 (17)	0.89463 (11)	−0.00442 (7)	0.1046 (5)	
O1	−0.1377 (2)	1.14174 (17)	0.30563 (13)	0.0563 (5)	
N1	0.3564 (2)	0.88167 (15)	0.23773 (11)	0.0350 (4)	
N2	0.0334 (3)	1.2760 (2)	0.33460 (15)	0.0547 (6)	
C1	0.1001 (3)	0.6176 (2)	0.40293 (14)	0.0424 (6)	
C2	0.0568 (4)	0.7187 (2)	0.42740 (14)	0.0464 (6)	
H2	−0.0033	0.7254	0.4677	0.056*	
C3	0.1046 (3)	0.8104 (2)	0.39085 (14)	0.0432 (5)	
H3	0.0799	0.8791	0.4080	0.052*	
C4	0.1888 (3)	0.80111 (17)	0.32911 (12)	0.0323 (4)	
C5	0.2270 (3)	0.69817 (19)	0.30503 (13)	0.0358 (5)	
H5	0.2818	0.6907	0.2634	0.043*	
C6	0.1841 (3)	0.60526 (19)	0.34250 (14)	0.0402 (5)	
H6	0.2122	0.5364	0.3267	0.048*	
C7	0.2364 (3)	0.90357 (17)	0.29068 (12)	0.0332 (5)	
H7	0.2824	0.9549	0.3241	0.040*	
C8	0.0906 (3)	0.95793 (18)	0.25622 (14)	0.0361 (5)	
H8A	0.0433	0.9093	0.2224	0.043*	
H8B	0.0099	0.9751	0.2907	0.043*	
C9	0.1484 (2)	1.06071 (18)	0.22138 (13)	0.0336 (5)	
C10	0.2759 (2)	1.03897 (18)	0.16737 (12)	0.0328 (4)	
H10	0.2308	0.9871	0.1341	0.039*	
C11	0.4174 (2)	0.98078 (18)	0.20494 (13)	0.0326 (4)	
H11	0.4597	1.0292	0.2409	0.039*	
C12	0.5523 (2)	0.95166 (19)	0.15563 (13)	0.0346 (5)	
C13	0.6907 (3)	1.0149 (2)	0.15428 (18)	0.0477 (6)	
H13	0.7032	1.0707	0.1864	0.057*	
C14	0.8102 (3)	0.9962 (3)	0.1058 (2)	0.0583 (8)	
H14	0.9014	1.0398	0.1047	0.070*	
C15	0.7925 (4)	0.9128 (3)	0.05980 (18)	0.0587 (8)	
C16	0.6613 (4)	0.8458 (3)	0.06195 (18)	0.0600 (8)	
H16	0.6530	0.7874	0.0315	0.072*	
C17	0.5407 (3)	0.8655 (2)	0.10996 (16)	0.0470 (6)	
H17	0.4512	0.8204	0.1114	0.056*	
C18	0.3314 (3)	1.1388 (2)	0.12639 (14)	0.0401 (5)	
H18A	0.3625	1.1955	0.1586	0.048*	
H18B	0.4255	1.1194	0.0995	0.048*	
C19	0.2027 (4)	1.1833 (2)	0.07772 (17)	0.0519 (7)	
H19A	0.2440	1.2455	0.0535	0.078*	
H19B	0.1731	1.1282	0.0449	0.078*	
H19C	0.1100	1.2043	0.1041	0.078*	
C20	0.1091 (3)	1.16038 (19)	0.24254 (13)	0.0368 (5)	
H20	0.1610	1.2180	0.2208	0.044*	
C21	−0.0092 (3)	1.18930 (19)	0.29731 (14)	0.0393 (5)	
H1N	0.430 (3)	0.850 (3)	0.2575 (17)	0.050 (9)*	
H2NA	−0.032 (3)	1.298 (3)	0.3633 (15)	0.047 (9)*	
H2NB	0.125 (2)	1.301 (3)	0.334 (2)	0.066 (11)*	

### Atomic displacement parameters (Å^2^)

**Table:**

	*U*^11^	*U*^22^	*U*^33^	*U*^12^	*U*^13^	*U*^23^
Cl1	0.1153 (8)	0.0524 (4)	0.0536 (4)	−0.0239 (4)	0.0060 (5)	0.0144 (3)
Cl2	0.1058 (8)	0.1019 (8)	0.1062 (9)	0.0398 (7)	0.0651 (8)	0.0203 (7)
O1	0.0416 (10)	0.0438 (10)	0.0835 (16)	0.0000 (8)	0.0188 (10)	−0.0017 (10)
N1	0.0271 (9)	0.0326 (9)	0.0453 (11)	0.0044 (7)	−0.0004 (8)	0.0075 (8)
N2	0.0546 (14)	0.0479 (13)	0.0615 (16)	−0.0027 (11)	0.0157 (13)	−0.0105 (12)
C1	0.0502 (14)	0.0401 (12)	0.0370 (13)	−0.0066 (10)	−0.0053 (11)	0.0088 (10)
C2	0.0533 (14)	0.0520 (15)	0.0339 (12)	0.0017 (12)	0.0073 (11)	0.0025 (10)
C3	0.0492 (14)	0.0391 (12)	0.0413 (13)	0.0082 (10)	0.0035 (11)	−0.0012 (10)
C4	0.0303 (9)	0.0321 (10)	0.0345 (11)	0.0019 (8)	−0.0046 (9)	0.0032 (8)
C5	0.0326 (10)	0.0372 (11)	0.0375 (12)	−0.0005 (9)	0.0011 (9)	−0.0016 (9)
C6	0.0454 (13)	0.0317 (11)	0.0436 (13)	−0.0005 (9)	−0.0047 (11)	−0.0017 (9)
C7	0.0315 (10)	0.0293 (10)	0.0387 (12)	−0.0006 (8)	−0.0021 (9)	0.0009 (8)
C8	0.0265 (9)	0.0305 (10)	0.0514 (14)	0.0017 (8)	0.0029 (9)	0.0078 (9)
C9	0.0247 (9)	0.0319 (10)	0.0443 (12)	0.0013 (8)	−0.0018 (9)	0.0068 (9)
C10	0.0271 (9)	0.0308 (9)	0.0406 (12)	0.0001 (8)	−0.0003 (8)	0.0019 (9)
C11	0.0254 (9)	0.0326 (10)	0.0399 (12)	−0.0016 (8)	−0.0016 (8)	−0.0008 (9)
C12	0.0280 (9)	0.0342 (10)	0.0416 (12)	0.0036 (8)	−0.0007 (9)	0.0041 (9)
C13	0.0318 (11)	0.0512 (14)	0.0602 (16)	−0.0026 (10)	0.0024 (12)	−0.0017 (13)
C14	0.0344 (13)	0.0653 (19)	0.075 (2)	0.0031 (12)	0.0135 (13)	0.0118 (16)
C15	0.0528 (16)	0.0614 (18)	0.0619 (18)	0.0242 (14)	0.0213 (14)	0.0157 (14)
C16	0.079 (2)	0.0479 (15)	0.0534 (17)	0.0151 (15)	0.0114 (16)	−0.0061 (12)
C17	0.0484 (14)	0.0404 (12)	0.0522 (16)	0.0003 (11)	0.0026 (12)	−0.0026 (11)
C18	0.0355 (11)	0.0383 (11)	0.0465 (14)	0.0017 (9)	0.0071 (10)	0.0073 (10)
C19	0.0494 (15)	0.0527 (15)	0.0537 (16)	0.0108 (12)	0.0062 (13)	0.0176 (13)
C20	0.0338 (10)	0.0308 (10)	0.0457 (13)	0.0001 (8)	0.0032 (10)	0.0057 (9)
C21	0.0387 (12)	0.0299 (11)	0.0494 (14)	0.0060 (9)	0.0053 (10)	0.0065 (9)

### Geometric parameters (Å, º)

**Table:**

Cl1—C1	1.742 (3)	C9—C20	1.332 (3)
Cl2—C15	1.752 (3)	C9—C10	1.513 (3)
O1—C21	1.230 (3)	C10—C18	1.531 (3)
N1—C7	1.455 (3)	C10—C11	1.558 (3)
N1—C11	1.464 (3)	C10—H10	0.9800
N1—H1N	0.821 (14)	C11—C12	1.516 (3)
N2—C21	1.334 (4)	C11—H11	0.9800
N2—H2NA	0.824 (14)	C12—C17	1.381 (4)
N2—H2NB	0.824 (14)	C12—C13	1.390 (3)
C1—C6	1.370 (4)	C13—C14	1.386 (4)
C1—C2	1.378 (4)	C13—H13	0.9300
C2—C3	1.389 (4)	C14—C15	1.365 (5)
C2—H2	0.9300	C14—H14	0.9300
C3—C4	1.388 (4)	C15—C16	1.370 (5)
C3—H3	0.9300	C16—C17	1.389 (4)
C4—C5	1.385 (3)	C16—H16	0.9300
C4—C7	1.515 (3)	C17—H17	0.9300
C5—C6	1.399 (3)	C18—C19	1.528 (4)
C5—H5	0.9300	C18—H18A	0.9700
C6—H6	0.9300	C18—H18B	0.9700
C7—C8	1.538 (3)	C19—H19A	0.9600
C7—H7	0.9800	C19—H19B	0.9600
C8—C9	1.510 (3)	C19—H19C	0.9600
C8—H8A	0.9700	C20—C21	1.489 (4)
C8—H8B	0.9700	C20—H20	0.9300
			
C7—N1—C11	112.88 (17)	C11—C10—H10	107.3
C7—N1—H1N	106 (3)	N1—C11—C12	109.44 (18)
C11—N1—H1N	110 (2)	N1—C11—C10	108.71 (17)
C21—N2—H2NA	117 (2)	C12—C11—C10	112.1 (2)
C21—N2—H2NB	123 (3)	N1—C11—H11	108.8
H2NA—N2—H2NB	120 (4)	C12—C11—H11	108.8
C6—C1—C2	121.7 (2)	C10—C11—H11	108.8
C6—C1—Cl1	119.9 (2)	C17—C12—C13	118.3 (2)
C2—C1—Cl1	118.4 (2)	C17—C12—C11	122.1 (2)
C1—C2—C3	118.9 (2)	C13—C12—C11	119.6 (2)
C1—C2—H2	120.6	C14—C13—C12	121.0 (3)
C3—C2—H2	120.6	C14—C13—H13	119.5
C4—C3—C2	121.0 (2)	C12—C13—H13	119.5
C4—C3—H3	119.5	C15—C14—C13	119.1 (3)
C2—C3—H3	119.5	C15—C14—H14	120.4
C5—C4—C3	118.7 (2)	C13—C14—H14	120.4
C5—C4—C7	122.3 (2)	C14—C15—C16	121.2 (3)
C3—C4—C7	119.0 (2)	C14—C15—Cl2	118.8 (3)
C4—C5—C6	120.9 (2)	C16—C15—Cl2	119.9 (3)
C4—C5—H5	119.6	C15—C16—C17	119.5 (3)
C6—C5—H5	119.6	C15—C16—H16	120.3
C1—C6—C5	118.8 (2)	C17—C16—H16	120.3
C1—C6—H6	120.6	C12—C17—C16	120.7 (3)
C5—C6—H6	120.6	C12—C17—H17	119.7
N1—C7—C4	111.75 (18)	C16—C17—H17	119.7
N1—C7—C8	108.56 (19)	C19—C18—C10	113.2 (2)
C4—C7—C8	111.50 (18)	C19—C18—H18A	108.9
N1—C7—H7	108.3	C10—C18—H18A	108.9
C4—C7—H7	108.3	C19—C18—H18B	108.9
C8—C7—H7	108.3	C10—C18—H18B	108.9
C9—C8—C7	107.72 (18)	H18A—C18—H18B	107.8
C9—C8—H8A	110.2	C18—C19—H19A	109.5
C7—C8—H8A	110.2	C18—C19—H19B	109.5
C9—C8—H8B	110.2	H19A—C19—H19B	109.5
C7—C8—H8B	110.2	C18—C19—H19C	109.5
H8A—C8—H8B	108.5	H19A—C19—H19C	109.5
C20—C9—C8	123.6 (2)	H19B—C19—H19C	109.5
C20—C9—C10	123.1 (2)	C9—C20—C21	126.9 (2)
C8—C9—C10	112.56 (19)	C9—C20—H20	116.6
C9—C10—C18	115.28 (18)	C21—C20—H20	116.6
C9—C10—C11	106.90 (18)	O1—C21—N2	122.7 (3)
C18—C10—C11	112.36 (18)	O1—C21—C20	123.7 (2)
C9—C10—H10	107.3	N2—C21—C20	113.5 (2)
C18—C10—H10	107.3		
			
C6—C1—C2—C3	−2.2 (4)	C7—N1—C11—C10	−62.6 (2)
Cl1—C1—C2—C3	178.2 (2)	C9—C10—C11—N1	57.4 (2)
C1—C2—C3—C4	2.7 (4)	C18—C10—C11—N1	−175.2 (2)
C2—C3—C4—C5	−1.1 (4)	C9—C10—C11—C12	178.48 (18)
C2—C3—C4—C7	179.2 (2)	C18—C10—C11—C12	−54.1 (3)
C3—C4—C5—C6	−1.0 (3)	N1—C11—C12—C17	45.3 (3)
C7—C4—C5—C6	178.6 (2)	C10—C11—C12—C17	−75.4 (3)
C2—C1—C6—C5	0.1 (4)	N1—C11—C12—C13	−136.7 (2)
Cl1—C1—C6—C5	179.7 (2)	C10—C11—C12—C13	102.6 (3)
C4—C5—C6—C1	1.5 (4)	C17—C12—C13—C14	3.6 (4)
C11—N1—C7—C4	−173.73 (18)	C11—C12—C13—C14	−174.5 (3)
C11—N1—C7—C8	62.9 (2)	C12—C13—C14—C15	−1.3 (5)
C5—C4—C7—N1	−14.2 (3)	C13—C14—C15—C16	−1.9 (5)
C3—C4—C7—N1	165.5 (2)	C13—C14—C15—Cl2	176.3 (2)
C5—C4—C7—C8	107.5 (3)	C14—C15—C16—C17	2.7 (5)
C3—C4—C7—C8	−72.8 (3)	Cl2—C15—C16—C17	−175.5 (2)
N1—C7—C8—C9	−58.3 (2)	C13—C12—C17—C16	−2.8 (4)
C4—C7—C8—C9	178.2 (2)	C11—C12—C17—C16	175.2 (3)
C7—C8—C9—C20	−111.6 (2)	C15—C16—C17—C12	−0.3 (5)
C7—C8—C9—C10	59.3 (3)	C9—C10—C18—C19	−68.9 (3)
C20—C9—C10—C18	−13.2 (3)	C11—C10—C18—C19	168.3 (2)
C8—C9—C10—C18	175.8 (2)	C8—C9—C20—C21	−7.2 (4)
C20—C9—C10—C11	112.5 (2)	C10—C9—C20—C21	−177.2 (2)
C8—C9—C10—C11	−58.5 (2)	C9—C20—C21—O1	−39.1 (4)
C7—N1—C11—C12	174.66 (19)	C9—C20—C21—N2	144.3 (3)

### Hydrogen-bond geometry (Å, º)

Cg is the centroid of the C1–C6 ring.

**Table:**

*D*—H···*A*	*D*—H	H···*A*	*D*···*A*	*D*—H···*A*
N2—H2*NB*···O1^i^	0.82 (1)	2.17 (2)	2.973 (3)	165 (4)
C6—H6···O1^ii^	0.93	2.55	3.454 (3)	163
N1—H1*N*···*Cg*^ii^	0.82 (1)	2.85 (4)	3.626 (2)	157 (3)

Symmetry codes: (i) *x*+1/2, −*y*+5/2, *z*; (ii) *x*+1/2, −*y*+3/2, *z*.
