# Supplementary figures and images for: Crystal structure of 2-[4(E)-2,6-bis­(4-chloro­phen­yl)-3-ethyl­piperidin-4-yl­idene]acetamide
Source: Acta Crystallogr E Crystallogr Commun. 2015 Oct 10;71(Pt 11):o832–3. doi: 10.1107/S2056989015018666 (PMC4645048; doi:10.1107/S2056989015018666)

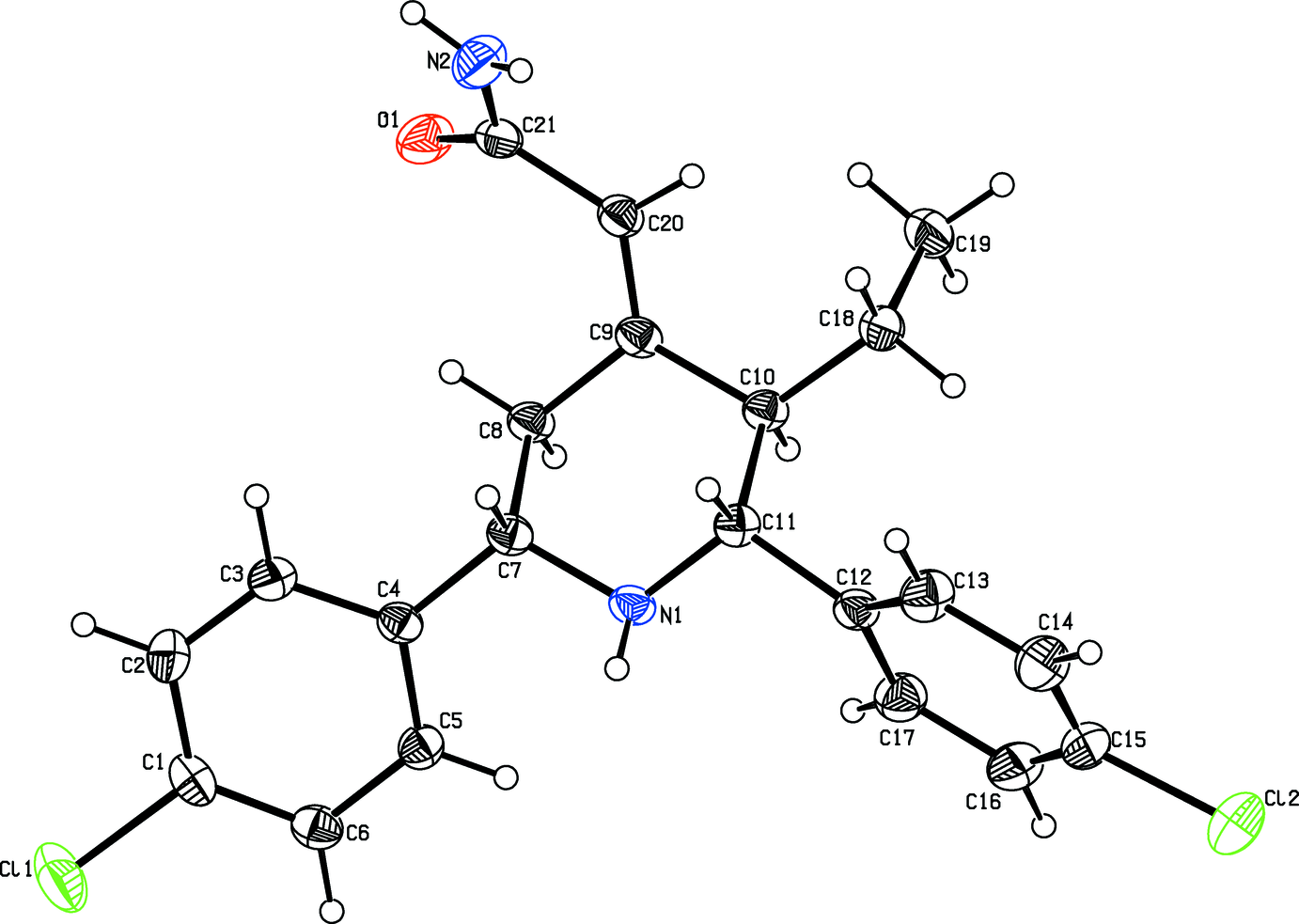

Supplement: Supplementary file 3 [file e-71-0o832-fig1.tif]

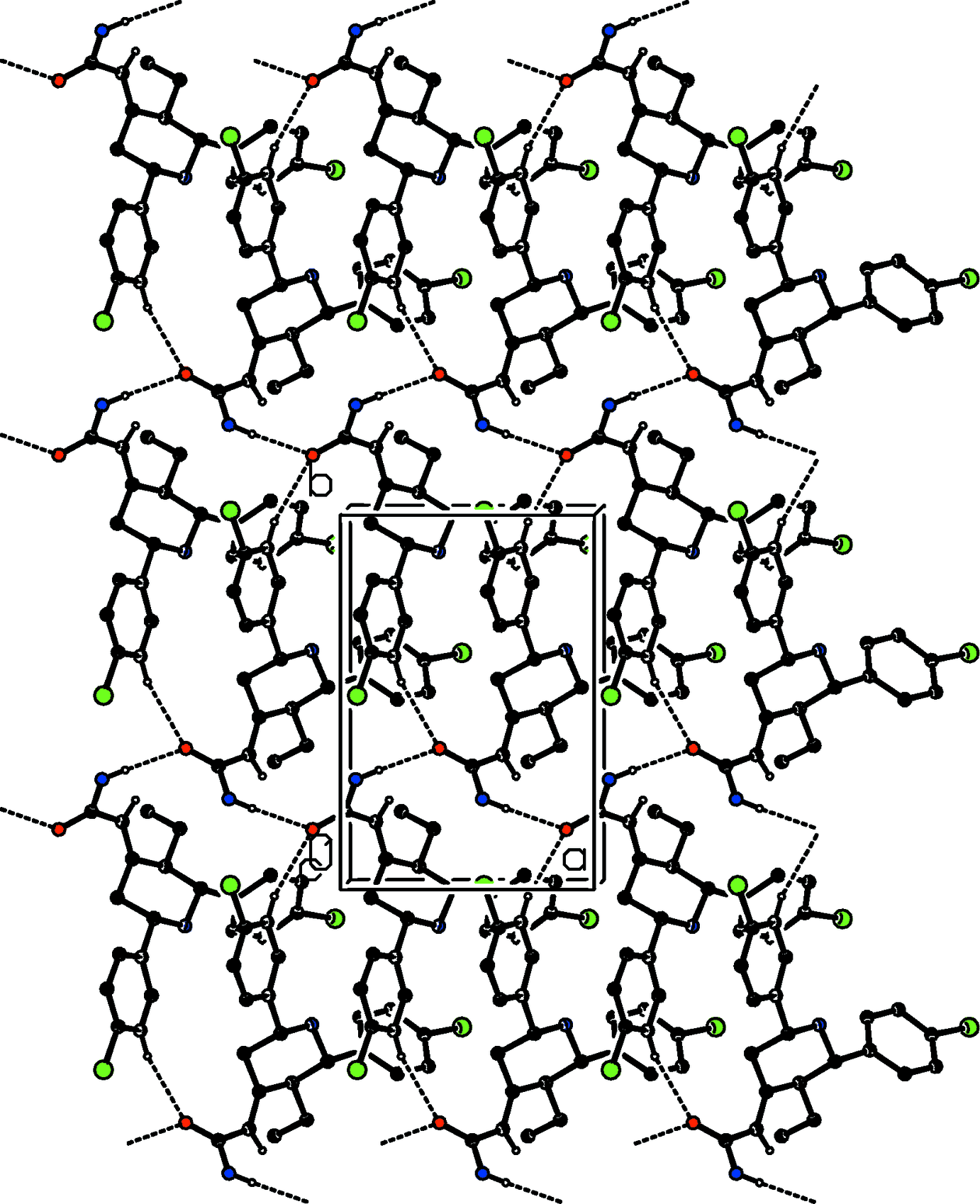

Supplement: Supplementary file 4 [file e-71-0o832-fig2.tif]

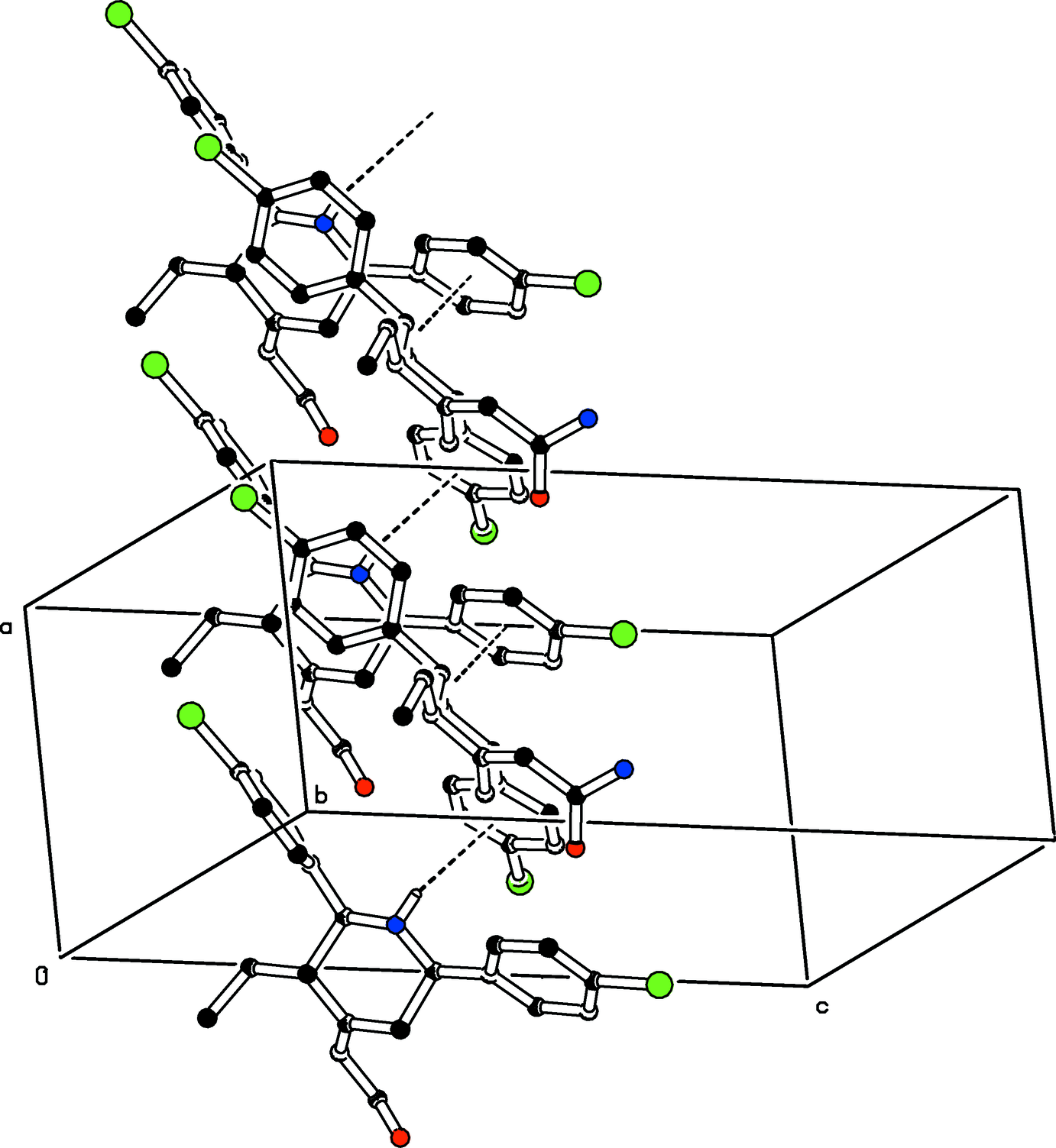

Supplement: Supplementary file 5 [file e-71-0o832-fig3.tif]
